# Hepatocyte-specific *Smad7* deletion accelerates DEN-induced HCC via activation of STAT3 signaling in mice

**DOI:** 10.1038/oncsis.2016.85

**Published:** 2017-01-30

**Authors:** T Feng, J Dzieran, X Yuan, A Dropmann, T Maass, A Teufel, S Marhenke, T Gaiser, F Rückert, I Kleiter, S Kanzler, M P Ebert, A Vogel, P ten Dijke, S Dooley, N M Meindl-Beinker

**Affiliations:** 1 Department of Medicine II, Section Molecular Hepatology – Alcohol Associated Diseases, Medical Faculty Mannheim, Heidelberg University, Mannheim, Germany; 2 Department of Internal Medicine I, University Hospital Regensburg, Regensburg, Germany; 3 Department of Gastroenterology, Hepatology and Endocrinology, Medical School Hannover, Hannover, Germany; 4 Institute of Pathology, University Medical Center Mannheim, Medical Faculty Mannheim, Heidelberg University, Mannheim, Germany; 5 Department of Surgery, University Medical Centre Mannheim, Medical Faculty Mannheim, Heidelberg University, Mannheim, Germany; 6 Department of Neurology, St Josef-Hospital, Ruhr-University Bochum, Bochum, Germany; 7 Department of Internal Medicine 2, Leopoldina-Hospital Schweinfurt, Schweinfurt, Germany; 8 Department of Medicine II, Universitätsmedizin Mannheim, Medical Faculty Mannheim, Heidelberg University, Mannheim, Germany; 9 Department of Molecular Cell Biology, Cancer Genomics Centre Netherlands, Leiden University Medical Center, RC Leiden, The Netherlands

## Abstract

TGF-β signaling in liver cells has variant roles in the dynamics of liver diseases, including hepatocellular carcinoma (HCC). We previously found a correlation of high levels of the important endogenous negative TGF-β signaling regulator *SMAD7* with better clinical outcome in HCC patients. However, the underlying tumor-suppressive molecular mechanisms are still unclear. Here, we show that conditional (TTR-Cre) hepatocyte-specific SMAD7 knockout (KO) mice develop more tumors than wild-type and corresponding SMAD7 transgenic mice 9 months after diethylnitrosamine (DEN) challenge, verifying SMAD7 as a tumor suppressor in HCC. In line with our findings in patients, *Smad7* levels in both tumor tissue as well as surrounding tissue show a significant inverse correlation with tumor numbers. SMAD7 KO mice presented with increased pSMAD2/3 levels and decreased apoptosis in the tumor tissue. Higher tumor incidence was accompanied by reduced P21 and upregulated c-MYC expression in the tumors. Activation of signal transducer and activator of transcription factor 3 signaling was found in *Smad7*-deficient mouse tumors and in patients with low tumoral *SMAD7* expression as compared with surrounding tissue. Together, our results provide new mechanistic insights into the tumor-suppressive functions of SMAD7 in hepatocarcinogenesis.

## Introduction

Hepatocellular carcinoma (HCC) is the most frequent primary liver tumor in humans.^1^ Transforming growth factor-β (TGF-β) expression is generally upregulated in most chronic liver disease (CLDs) and its signaling has important roles in different aspects, including fibrogenesis, immune modulation and progression of HCC.^2^ A dual role of TGF-β signaling in CLD has been well documented.^3^ In the early stages of liver disease and HCC development, TGF-β exhibits cytostatic effects as apoptosis induction and proliferation inhibition towards liver epithelial cells. In late disease stages, it promotes epithelial-to-mesenchymal transition, cell migration and invasion and thus facilitates tumor development and progression. As a major negative regulator of TGF-β family signaling, Sma- and Mad-related protein 7 (SMAD7) was consequently found to have both tumor-promotive as well as -suppressive functions not only in HCC but also in different types of cancer, in variant cell types and at different stages of disease dynamics. On the one hand, SMAD7 can interfere with TGF-β signaling and therefore can change its roles in the process of tumor development. Its effect on tumorigenesis is then directly dependent on the respective spatio-temporal effects of TGF-β. On the other hand, SMAD7 can have TGF-β-independent functions, for example, can interact with several proteins that are not related to TGF-β signaling, thus affecting tumor progression non-canonically.^4^ In human HCC, Wang *et al.*^5^ and Xia *et al.*^6^ showed reduced *SMAD7* levels in tumor tissue. However, in a larger patient cohort, our group recently described that *SMAD7* in the mean is increased in tumor tissue compared with the surrounding tissue.^7^ These differences reflect the heterogeneity of HCC and, as discussed in Feng *et al.*,^7^ are probably due to different staging of the HCC, the grade of underlying cirrhosis or fibrosis and other patient characteristics subclassifying the cohorts analyzed. Although *SMAD7* was conversely regulated in the mean of both the above cohorts, samples with *SMAD7* upregulation and others with downregulation were included in each of them reflecting the vast number of other factors relevant for HCC progression and the necessity of an individualized investigation. Regardless of the *SMAD7* distribution in different patient cohorts, Xia *et al.*^6^ concurred with our results that high *SMAD7* levels in tumor tissue correlate with better survival of the patients.

Owing to the complex ambivalent nature of TGF-β/SMAD7 signaling, knowledge about the mechanistic role of SMAD7 in HCC is still limited. To fill this gap, different mouse models were established in the past. In the early stages of liver damage in mice induced by fumarylacetoacetase deficiency, we have shown that SMAD7 can regulate compensatory hepatocyte proliferation by directly inhibiting TGF-β cytostatic effects and apoptosis control.^7^ Wang *et al.*^5^ in comparison reported that SMAD7 acts as tumor suppressor in mice that are constitutively expressing a truncated SMAD7 protein in diethylnitrosamine (DEN)-induced HCC. Although liver disease-associated TGF-β signaling is exhibited by all different cell types in the liver, such as stellate cells, Kupffer cells and hepatocytes,^2^ HCC was recently reported to arise exclusively from hepatocytes.^8^ Thus, we decided to further evaluate the functional role and mechanistic background of SMAD7 in DEN-induced mouse HCC by hepatocyte-specific gain and loss of SMAD7 expression. We established Tamoxifen-inducible hepatocyte-specific (albumin promoter) SMAD7 transgenic (Tg) and knockout (KO) mouse models, which both displayed a patient-like patchy differential SMAD7 expression in the liver tissue.

Signal transducer and activator of transcription factor 3 (STAT3) signaling in HCC progression and its link to inflammatory mechanisms was nicely reviewed by He and Karin.^9^ STAT3 is a transcription factor that is activated upon different cytokine stimulations such as interleukin (IL)-5 or epidermal growth factor, and profound activation was demonstrated in HCC.^10, 11, 12^ A link to aggressive behavior and malignant transformation of progenitor cells was also delineated.^13^ Mechanistically, events leading to STAT3 activation in HCC are not fully understood; however, it is known that activating mutations are rare. Thus, activation is probably induced by exogenous or endogenous factors, for example, expressed in the tumor microenvironment considering IL-6, IL-22 and/or IL-11 to be most apparent regulators.^9^ In addition, the STAT3 activating or deactivating role of oxidative stress is discussed, and regulation of nuclear factor (NF)-κB and STAT3 seem to be oppositional.^10, 14^ Mishra and co-workers interestingly demonstrated that, especially in cells with inactive TGF-β signaling, inhibition of STAT3 activation would provide an effective approach in the management of HCCs.^12^ In HCC cell lines with reduced levels of TGF-β pathway proteins and in isolated liver cancer stem cells with diminished TGF-β type II receptor expression, as well as *in vivo* using a xenograft model, the STAT3 inhibitor NSC 74859 was able to diminish cell proliferation and/or tumor growth more effectively than in cellular systems with intact TGF-β signaling.

Building on these results, we found for the first time a link between induced STAT3 signaling and hepatocyte-specific SMAD7 deficiency in DEN mice and human HCC, thus unraveling a new tumor-suppressive mechanism for SMAD7 in HCC.

## Results

### HCC induction in TTR-Cre hepatocyte-specific SMAD7 Tg and KO mice

**Figure 1:**
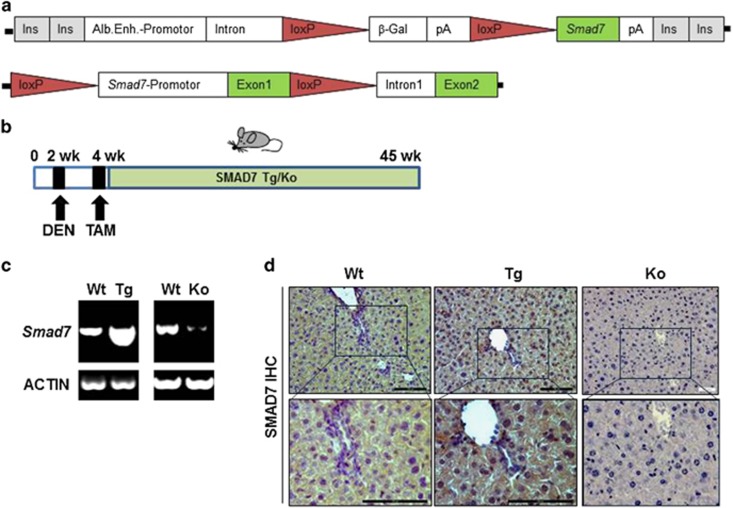
DEN-dependent HCC in Tamoxifen (TAM)-inducible hepatocyte-specific SMAD7 Tg and SMAD7 KO mice. (**a**) Plasmid constructs used for generating Albumin-SMAD7 Tg and SMAD7^fl/fl^ mice. (**b**) Experimental design of the DEN-induced HCC in TTR-Cre × SMAD7 Tg or KO mice. (**c**) *Smad7* levels were analyzed by RT–PCR and (**d**) immunohistochemistry (positive staining is indicated by brown color).

In order to understand the molecular mechanism of SMAD7 as a tumor suppressor in the development of HCC, Tamoxifen-inducible hepatocyte-specific SMAD7 expression was induced or blunted using albumin promoter-driven constructs in SMAD7 Tg and SMAD7 KO mice (Figures 1a and b). Three months after injecting Tamoxifen, DNA recombination was monitored at the DNA level in both, SMAD7 Tg and KO mice (Supplementary Figure S1A). Liver parameters were not altered by Tamoxifen treatment and subsequent SMAD7 expression modulation (Supplementary Figure S1B). Changes of *Smad7* mRNA levels were analyzed by reverse transcriptase–PCR (RT–PCR) (Figure 1c) and confirmed increased or reduced *Smad7* expression in SMAD7 Tg or SMAD7 KO mice. Overexpressed SMAD7 was additionally detected by immunohistochemistry (Figure 1d).

**Figure 2:**
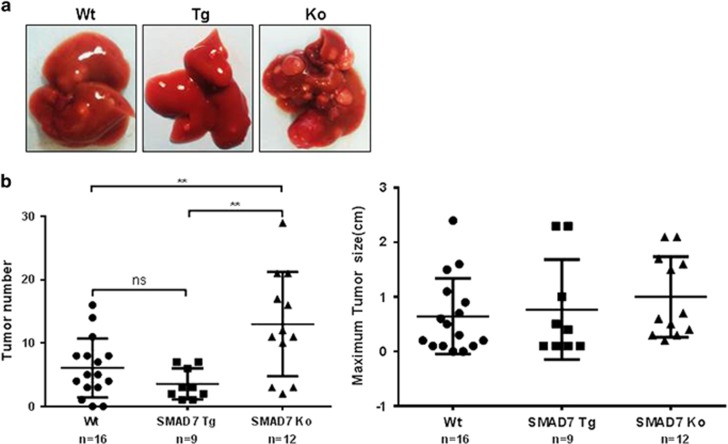
Hepatocyte-pecific SMAD7 deletion accelerates DEN-induced HCC development in mice. (**a**) Representative liver images from WT, SMAD7 Tg and KO mice after DEN treatment. (**b**) SMAD7 KO mice showed significantly more tumors compared with WT and SMAD7 Tg mice, as calculated by one-way analysis of variance test. ns, not significant. **P⩽0.01.

Induction of HCC by DEN treatment was confirmed by histopathological analyses of hematoxylin and eosin (H&E)-stained slides. Representative photos of the livers of wild-type (WT), Tg and KO animals and H&E-stained tissue sections are shown in Figure 2a and as Supplementary Figure S2A. SMAD7 KO animals developed significantly more tumors than WT or SMAD7 Tg mice (*P*≤0.01), whereas the size of tumors was not altered in the mean (Figure 2b). Liver weights and liver/body weight ratios did not differ between WT, SMAD7 Tg and SMAD7 KO mice (Supplementary Figures S2B and C). Together, these results characterized SMAD7 as a tumor suppressor in our model of DEN-induced HCC in mice, which is in accordance to our findings in human patient cohorts.^7^ The data also indicate that tumor initiation rather than tumor growth was effected by SMAD7 expression modulation.

### Correlation between SMAD7 levels and tumor number

**Figure 3:**
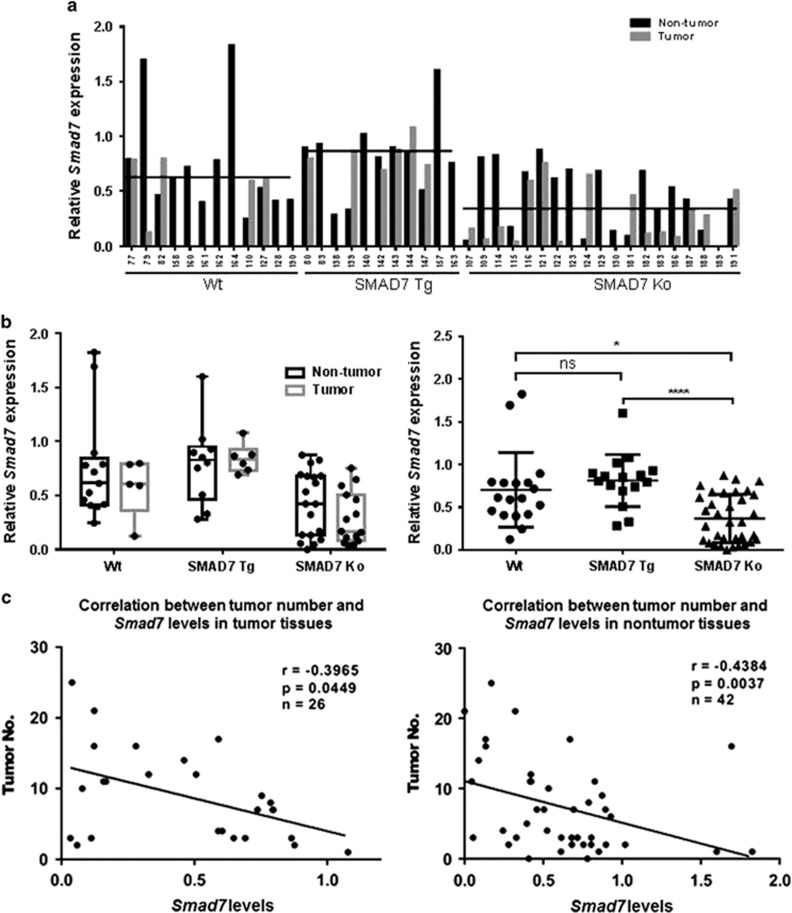
*Smad7* levels in both tumor tissue and surrounding tissue negatively correlate with tumor numbers. (**a**, **b**) Densitometric analysis of *Smad7* levels based on RT–PCR results (Supplementary Figure S2) revealed significant *Smad7* downregulation in SMAD7 KO animals and by trend upregulation in SMAD7 Tg animals. (**c**) Negative correlation between *Smad7* levels and tumor numbers in tumor tissue (*P*=0.0449), as well as in surrounding tissue (*P*=0.0037) (Spearman's rank-order coefficient test) was detected. ns, not significant. *P≤0.05, ****P≤0.0001.

Although the conditional Tamoxifen-dependent gene expression system is a useful tool in biological research,^15^ the transgene induction efficiency by Tamoxifen varies depending on species, tissue type and individual animals.^16^ In line, we found mice with no or little response to Tamoxifen with regard to SMAD7 overexpression/depletion in our study (Supplementary Figure S3). TGF-β signaling occurs not only in tumor cells but also in surrounding non-tumorigenic epithelial cells and the tumor stroma.^17^ We have shown recently that low *SMAD7* in both tumor tissue and surrounding tissue correlates with worse patient outcome.^7^ Similarly, *Smad7* levels in the present model varied, when comparing different tissue areas of the same animal, independent if Tg or KO genotypes were existent (Supplementary Figure S3), thus verifying our ‘leaky' model as a powerful tool to experimentally mimic human patient characteristics. Interestingly, *Smad7* was not generally upregulated or downregulated in tumor or surrounding tissue areas, but the expression was found to be high as well as low both in tumor as well as non-tumor tissue of different animals. In line, we have previously shown in patients that expression ratios between tumors and tumor surrounding tissues cover the range between ~0.08- and 38-fold. However, in average *Smad7* in the liver was significantly downregulated in SMAD7 KO and by trend upregulated in SMAD7 Tg mice as compared with WT (Figures 3a and b).

We next correlated *Smad7* mRNA levels detected by RT–PCR in tumors or surrounding tissue of mouse livers with tumor numbers in the respective animals, regardless of the underlying mouse genotype or the expression ratio of *Smad7* in tumors vs surrounding tissue. As shown in Figure 3c, *Smad7* levels in tumors as well as in the surrounding tissue were negatively correlated with tumor numbers (*P*=0.0449 and *P*=0.0037, respectively). These data perfectly match our previous findings in patients, indicating a survival correlation with *SMAD7* expression levels in tumors or surrounding tissue, which was, however, independent of the *SMAD7* expression ratio in tumors vs surrounding tissue.^7^ This highlights changes in the microenvironment during the development of HCC and the importance of SMAD7 in both tumor and non-tumor cells in the tumorigenic process.

### SMAD7 interferes with TGF-β signaling in DEN-induced mouse HCC

**Figure 4:**
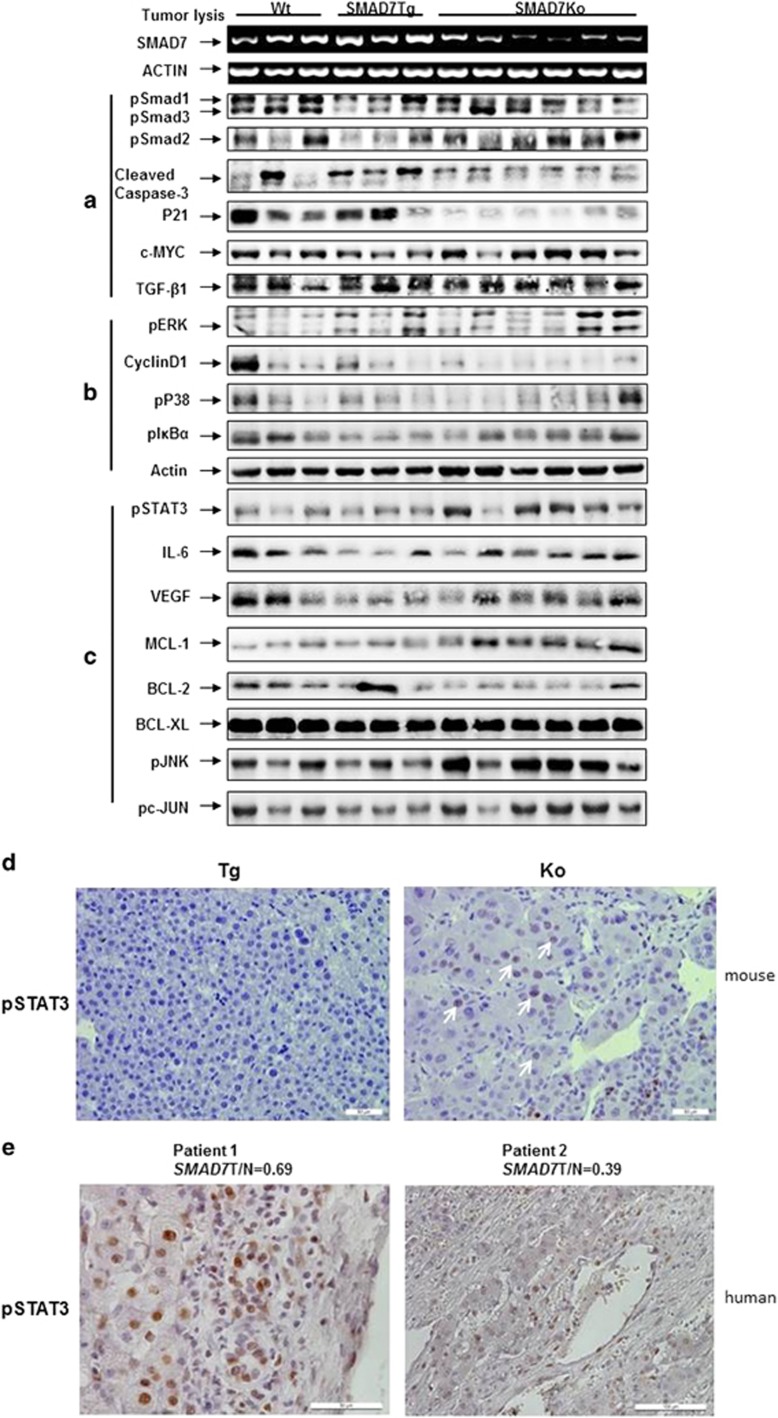
Activation of STAT3 signaling in tumors of SMAD7 KO mice. (**a**) SMAD7 overexpression or KO affects TGF-β signaling directly, as shown by modulation of Smad2/3 phosphorylation, but does not inhibit apoptosis and proliferation control as indicated by caspase 3 cleavage and P21 expression. (**b**) Crosstalk between TGF-β signaling and MAPK-ERK and P38, as well as NF-κB signaling was evaluated by immunoblot analysis. (**c**) Upregulation of IL-6, VEGF and MCL-1 expression, as well as the phosphoprotein levels of STAT3, JNK and c-JUN, occurs in tumor lysates of SMAD7 KO animals. For quantification of western blotting results, please see Supplementary Figure S4. (**d**) Positive IHC staining for pSTAT3 in tumor tissue of SMAD7 KO animals (indicated by white arrows), whereas tumors in TG animals stained negative. (**e**) Increased expression of pSTAT3 in human HCC tissue with low *SMAD7* expression compared with surrounding tissue.^21^ Patient 1 exhibits 0.69-fold and patient 2 0.39-fold *SMAD7* expression in HCC as compared with surrounding tissue.

Being confident to mimic the TGF-β/SMAD7 signaling status in human patients by the chosen mouse model, we first checked the impact of SMAD7 overexpression and KO on TGF-β signaling in the mouse tumors (Figure 4a). *Smad7* levels in individual mouse tumors were measured by RT–PCR; the amounts of other signaling components were determined by immunoblot and were quantified upon normalization to actin alone or actin and *Smad7* levels. We found increased levels of TGF-β1, pSMAD2 and pSMAD3 in SMAD7 KO mice, whereas levels were slightly reduced in tumors of SMAD7 Tg animals (Figure 4a, Supplementary Figure S4A), indicating that SMAD7 indeed negatively affects TGF-β signaling in our mouse model. As TGF-β is known to exhibit cytostatic functions in the liver, we also analyzed the expression of apoptotic and cell cycle regulatory proteins in the tumors. With respect to *Smad7* levels, c-MYC and cleaved caspase-3 expression was increased in tumors of SMAD7 KO mice. Quantification of immunoblot results also revealed upregulation of pERK related to survival signaling in SMAD7 KO animals. Interestingly, P21 was downregulated in tumors of SMAD7 KO animals (Figure 4a, Supplementary Figure S4B), suggesting that by deletion of SMAD7 cell cycle arrest is deteriorated. Although WT and Tg animals showed positive immunohistochemical staining of P21 in the liver tissue, especially in the non-tumor areas, KO animals did not exhibit significant P21 expression in either area (Supplementary Figure S5A). Interestingly, Ki67 staining was positive in WT and Tg mice, indicating proliferative activity in non-tumour and tumor areas, despite upregulated P21 expression. In contrast, SMAD7 KO mice showed no Ki67 staining (Supplementary Figure S5B) indicating, on the one hand, increased apoptosis but weakened cell cycle arrest and cell survival in SMAD7 KO animals but, on the other hand, compensatory proliferation in WT and Tg animals. Similarly, alternative and complex regulation of P21-dependent hepatocarcinogenesis dependent on the strength of the underlying disease were described before,^18^ arguing for a more detailed comparative analysis of P21 functions in DEN-treated SMAD7 Tg and KO mice in future studies.

### SMAD7 accelerates DEN induced mice HCC by activating STAT3 signaling

To further delineate the pathways affected by SMAD7 mediating its tumor-suppressive function, we next analyzed the components described to interact with SMAD7/TGF-β signaling or impact HCC development using the same tissue samples as above. We found increased pIκBα in SMAD7 KO mice (Figure 4b, Supplementary Figure S4) and additionally observed activation of cancerogenic proproliferative c-Jun N-terminal kinase (JNK) signaling^19^ in SMAD7 KO tumors. We then defined a link between SMAD7 expression and STAT3 signaling in liver cancer by detecting increased pSTAT3 expression in liver tissue of SMAD7 KO mice (Figures 4c and d, Supplementary Figure S4). Accordingly, we found reduced STAT3-activating IL-6 expression in SMAD7 Tg mice and relatively increased IL-6 in tumors and serum of SMAD7 KO mice (Figure 4c, Supplementary Figure S7A). From these data, we hypothesize that SMAD7 might inhibit production of IL-6, which results in decreased STAT3 activation and signaling. In line, we found increased expression of three STAT3 downstream targets VEGF (vascular endothelial growth factor), c-MYC and MCL-1 in SMAD7 KO mice compared with WT or Tg mice, indicating enhanced angiogenesis, proliferation and suppressed apoptosis mediated by the STAT3 pathway. In contrast, there was no change for BCL-2, which previously has also been described to be regulated by STAT3 signaling in other settings,^20^ further reflecting the complexity of signaling crosstalk in the context of HCC.

### Impact of SMAD7 on STAT3 signaling in human HCC

**Figure 5:**
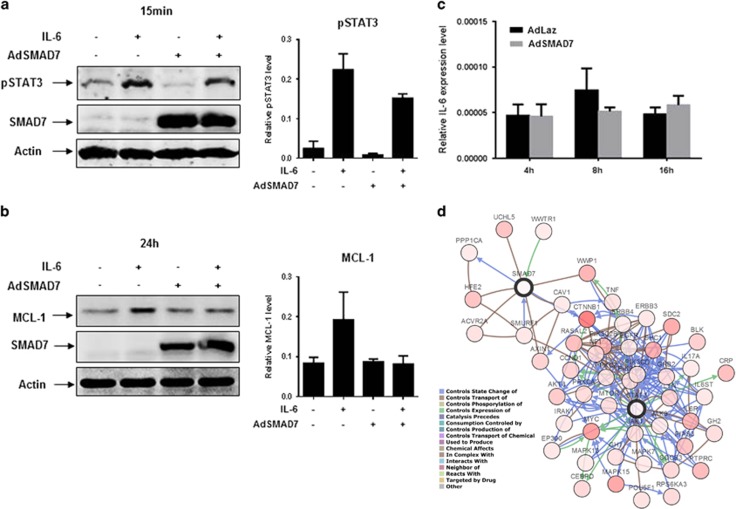
Adenovirus-mediated SMAD7 expression inhibits STAT3 phosphorylation in HuH-7. (**a**) Ectopic SMAD7 expression inhibits both endogenous and IL-6-induced STAT3 phosphorylation in HuH-7 cells. (**b**) Ectopic SMAD7 expression inhibits IL-6-induced MCL-1 expression in HuH-7 cells. Quantification of immunoblot analysis was performed of two independent experiments. One result is shown representatively. (**c**) *IL-6* expression levels were investigated by RT–PCR upon ectopic SMAD7 expression. (**d**) Theoretical pathway connections between SMAD7 and STAT3 in HCC analyzed by cBioPortal analysis (http://www.cbioportal.org/).

To functionally translate the expression correlations from the mouse model to human HCC cells, we next investigated whether *Smad7* expression in human HCC correlates with STAT3 activation (pSTAT3). Indeed, in three of the five human HCC samples, which we previously characterized to express low *SMAD7* in comparison to surrounding tissue,^21^ significant positive pSTAT3 staining was detected (Figure 4e, Supplementary Figure S6A). In contrast, no pSTAT3 was detected in tissue with high *SMAD7* expression ratios (Supplementary Figure S6B). To delineate a direct mechanistic link rather than correlation of SMAD7 and pSTAT3 activation, HuH-7 cells exhibiting autocrine IL-6/STAT3 signaling^22^ were infected with an adenovirus for SMAD7 overexpression. Upregulated SMAD7 inhibited intrinsic and IL-6-induced STAT3 phosphorylation in HuH-7 cells (Figure 5a) and inhibited IL-6-induced MCL-1 upregulation after 24 h in HuH-7 (Figure 5b). Consequently, TGF-β treatment induced pSTAT3 in HuH-7 cells, although the effect was much weaker than induction of pSTAT3 by IL-6 (Supplementary Figure S8), linking the SMAD7 effects on STAT3 signaling to TGF-β. In line with the expectation that cell types of the microenvironment such as macrophages rather than hepatocytes or tumor cells mainly produce IL-6, we could not find a significant effect of SMAD7 overexpression on the production of *Il-6* mRNA or secretion of IL-6 in HuH-7 cells (Figure 5c, Supplementary Figure S7B).

To stratify a possible link between STAT3, SMAD7 and canonical TGF-β signaling, we performed serial stainings of pSMAD2 and pSTAT3 in human HCC (Supplementary Figure S6). All patients with *SMAD7* T/N ratios <1 showed strong pSMAD2 positivity in the tumors, suggesting enhanced TGF-β signaling in tumors with low *SMAD7* levels, while STAT3 signaling was only activated in three patients and not all tumor cells. In one of these patients, pSTAT3 staining was restricted to only few cells. In patients with *SMAD7* T/N ratios >1, only one slide was positive for pSMAD2, indicating TGF-β signaling inhibition by high SMAD7, and absolutely no pSTAT3 staining was detected. However, these data do not finally resolve the question of a direct link between canonical TGF-β signaling, SMAD7 and STAT3.

Using the cBioPortal, we finally set up a possible network connection between SMAD7 and STAT3 in HCC.^23, 24^ Based on the integrative analysis of human HCC microarray data sets, multiple functional and mechanistic crosstalks between SMAD7 and STAT3 comprising signaling pathways were identified. In future, it will be of further interest which signaling outcomes (epithelial-to-mesenchymal transition, migration, invasion) the SMAD7/STAT3 axis directly effects to regulate hepatocellular carcinogenesis.

## Discussion

TGF-β signaling in CLD, HCC development and HCC progression is a broadly investigated field. Basically, TGF-β acts cytostatic toward the liver epithelium in acute liver damage, the regeneration process and early stages of CLD. At later stages of HCC development, TGF-β loses its cytostatic control ability and switches to a tumor-promoting effector. SMAD7, a TGF-β target gene, acts as a physiological feedback inhibitor of TGF-β family cytokines. Accordingly, SMAD7 might exhibit both tumor-suppressive or tumor-promotive effects, depending on spatial–temporal regulation of its biological action. Indeed, it has been described as a tumorigenic factor in the development of several tumors.^25^ In tissue samples of late-stage mouse HCC, we show in the present study that SMAD7 inhibits phosphorylation of SMAD2 and SMAD3 and exhibits inhibitory effects on distinct apoptotic and proliferation controlling pathways, including c-MYC and cleaved caspase-3, which have been associated with TGF-β signaling before.^26^ Based on its effect on tumor numbers, we classified SMAD7 as a tumor suppressor, in line with TGF-β-regulated non-canonical pathways such as p38 or pAKT and pERK, which were altered in tumors of KO mice.

Besides negatively regulating TGF-β signaling, tumor-suppressive functions might be due to crosstalks of SMAD7 with other signaling pathways, such as JNK, NF-κB and STAT3. All of them have been demonstrated to be important in the development of HCC before,^9, 10, 19^including DEN-induced mouse HCC.^27, 28, 29^ Accordingly, and in line with our data showing JNK activation and P21 reduction in SMAD7 KO tumors, it was described before that activation of proproliferative JNK signaling is a prerequisite of P21 downregulation and HCC cell proliferation in human tumor cells and in chemically induced mouse liver cancer.^19^ However, we could not confirm accompanying c-MYC downregulation. Generally, the effects of p21 on liver regeneration and hepatocarcinogenesis are dependent on the stage and severity of the underlying liver disease.^18^ Although loss of P21 in animals with severe liver injury promotes tumorigenesis, it impairs tumor development in animals with mild liver damage. In accordance, we demonstrated loss of tumor-suppressive P21 in late-stage liver disease of Smad7 KO mice while increased Ki67 expression described by Vogel and colleagues in severly injuried P21−/− mouse liver tissue^18^ was not detected in SMAD7 KO mice. Yet, it is not proven when in the course of tumorigenesis P21 was downregulated and which specific impact it had on different steps of cancer development.

Data from hepatocyte-specific STAT3 KO mice strongly suggested that activated STAT3 signaling is a predominant factor for HCC development in mice and association with human HCC was drawn.^10^ These data further suggest tumor-suppressive functions of IKKβ in hepatocytes. Consistently and in line with a previous report showing that SMAD7 inhibits mouse tumorigenesis by inhibiting NF-κB signaling,^30^ we found upregulation of pIκBα in SMAD7 overexpressing mouse tumor tissue. Profound activation of STAT3 signaling in SMAD7 KO mice and in patients with low SMAD7 expressing HCC, as shown here, now provides further evidence linking two important tumorigenic pathways in HCC, that is, TGF-β and STAT3. Autocrine IL-6 signaling is critical for liver cancer progenitor cell-related disease progression,^13^ suggesting future analysis of progenitor cell effects in SMAD7-dependent HCC. In the liver, IL-6 is mainly produced by macrophages or Kupffer cells at the beginning of tumor development,^27, 29^ although hepatocytes were also demonstrated to have the ability to produce IL-6 after challenge with lipopolysaccharide or hepatocyte growth factor.^31^ This might explain the clear effect of SMAD7 deletion in mouse tumor tissue on IL-6 expression, whereas the effect was only marginal in the autocrine-stimulated HuH-7 cell line.^22^ However, in line with former findings showing that SMAD7 suppresses IL-6 production in macrophages and CMT-93 tumor cells,^30^ we demonstrated that SMAD7 overexpression inhibits the phosphorylation of STAT3 as well as expression of the downstream target MCL-1 in HuH-7 cells, while SMAD7 knockdown in mice induced MCL-1 expression in the tumors. In contrast, Weber *et al.*^32^ showed compensatory hepatocyte proliferation and apoptosis induction, resulting in HCC development upon hepatocyte-specific deletion of MCL-1. These differences seen might be due to the etiology of HCC investigated (spontaneous vs DEN induced), which probably induces different sets of carcinogenic signaling events.^32^ These contrary data sets highlight once again the delicate balance between apoptosis, survival, regenerative and compensatory vs degenerated proliferation in liver diseases of different origins and stages. Dependent on the target cell and tissue as well as the specific signaling pathways used, every one of these cellular mechanisms can be advantageous or disadvantageous for the organ or the tumor. Thus, apoptosis of cancerous cells can, on the one hand, lead to tumor suppression; on the other hand, apoptosis can also induce compensatory proliferation leading to hepatocarcinogenesis.

In the future, several important questions remain to be further investigated, for example, whether SMAD7-dependent STAT3 regulation is TGF-β signaling mediated or an independent effect. Our immunohistochemical data suggest that SMAD7 levels do correlate with SMAD2 and STAT3 phosphorylation in human HCC. As staining of both targets, however, was not restricted to the same cells and tissue areas but pSMAD2 staining was usually spread across larger fields, we reason that SMAD7-dependent regulation of STAT3 signaling might not be exclusively dependent on canonical TGF-β signaling. However, as SMAD7 is a target gene of TGF-β itself, a link between TGF-β signaling strength and the impact of SMAD7 on STAT3 signaling is given. Accordingly, in HuH-7 cells SMAD7, on the one hand, was able to decrease pSTAT3 while, on the other hand, TGF-β induced STAT3 phosphorylation in TGF-β-sensitized HuH7 cells.

Other hints for a TGF-β-dependent link between SMAD7 and STAT3 signaling were provided before, although they usually focus on regulative effects of STAT3 on TGF-β signaling components and not vice versa. Thus, induction of SMAD7 expression by activated STAT3 signaling was shown to blunt TGF-β responses,^33, 34, 35^ and it was recently demonstrated that STAT3 signaling is able to inhibit TGF-β antiproliferative effects by interaction with SMAD3.^36^ How, on the other hand, negative regulation of TGF-β signaling by SMAD7, for example, in the context of HCC, influences STAT3 signaling reciprocally, is now of interest. In the chemoresistant colorectal cancer cell line DLD1-5FU-C10, SMAD3/4 inhibition negatively regulated STAT3 activation, suggesting existence of such reciprocal regulation between TGF-β and STAT3 signaling.^37^ However, TGF-β-independent tumor-suppressive mechanisms of SMAD7 are also very reasonable and would be in line with findings of Schwabe *et al.*,^38^ who recently demonstrated that epithelial TGF-β signaling rather is a protecting factor for mouse cholangiocarcinoma than a contributor to liver fibrosis and thus to subsequent HCC development. The findings provided by hepatocyte- and/or cholangiocyte-specific TGF-β receptor 2 and/or PTEN deletion in different mouse models of liver diseases might impact TGF-β-directed therapeutical options, as they probably have negative effects on the risk of cholangiocarcinogenesis in patients.^38^ Oxidative stress modulation was often described to integrate several key pathways in hepatocarcinogenesis.^14^ Thus, TGF-β-dependent and -independent effects of SMAD7 on STAT3, JNK and NF-κB signaling could be explained by a potential impact on the oxidative status of tumor cells and therefore needs to be investigated further.

**Figure 6:**
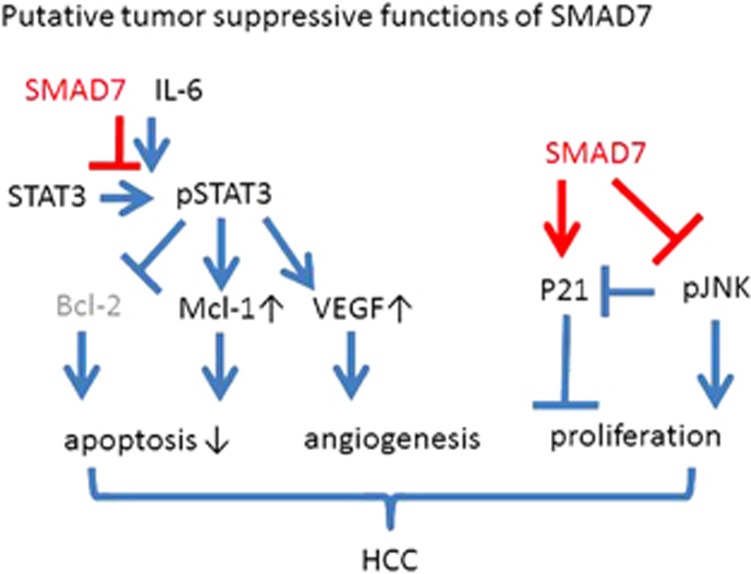
Scheme on tumor-suppressive functions of SMAD7 in DEN-induced mouse HCC. Summary of the tumor-suppressive effects of SMAD7 as delineated from the data obtained in DEN-induced HCC samples of SMAD7 Tg and SMAD7 KO mice. Target gene regulation was measured in tumor lysates of DEN-treated SMAD7 Tg and KO mice. Functional outcome of target gene regulation was based on literature (see Discussion section).

Taken together, our current study unveils a new mechanism with IL-6/STAT3 signaling as one target of tumor-suppressive SMAD7 effects in HCC as summarized in Figure 6. This ensures a deeper understanding of the pathological role of SMAD7 (and possibly TGF-β signaling) in the progression of HCC. Although many details of the mechanistic integration and crosstalk of this new pathway with the canonical SMAD7/TGF-β signaling and its functional switch in CLD progression remain open, it strengthens the tumor-suppressive role of SMAD7 in late-stage disease. If regulatory control of SMAD7 is missing at this stage, TGF-β exhibits increased tumor-promotive functions such as epithelial-to-mesenchymal transition, migration and invasion and the activation of tumor-promotive STAT3 signaling could further accelerate the vicious development. Next investigations need to delineate how and at which time point during disease progression the SMAD7/STAT3 route is initiated and whether this is indeed part of the functional switch of TGF-β/SMAD7 in liver tumorigenesis.

Further, it will be important to delineate the mechanism how in detail SMAD7 interferes with STAT3 signaling. We hypothesize that SMAD7 might have a role in STAT3/pSTAT3 protein degradation process, for example, via ubiquitinylation similar as it does with the activated TGF-beta receptor complex^39, 40^ or that binding of SMAD7 to STAT3 could suppress phosphorylation and thus activation for conformational reasons. However, initial immunoprecipitation experiments provided no indication for direct interaction of SMAD7 and STAT3 in HuH7 cells (data not shown). Further, array data analyses of AdSmad7-infected mouse hepatocytes revealed no significant SMAD7-dependent upregulation of canonical STAT3 upstream components, such as *gp130(Il6st)* or *Jak2* mRNA (data not shown). In summary, the data suggest that SMAD7 controls STAT3 activation and downstream signaling more indirectly, for example, by additional cellular components or by crosstalk of other signaling pathways yet to be identified.

As STAT3 signaling is important for the development of HCC, it is an attractive target for HCC treatment.^41^ Accordingly, a small molecular compound that can inhibit STAT3 signaling is now under clinical evaluation.^42^ The group of Mishra also demonstrated that targeting the IL-6/STAT3 pathway especially in association with an inhibition of the TGF-β/β2 spectrin pathway results in reduced proliferation of HCC cell lines and tumor progression in xenograft mice.^12^ The link between endogenously blunted TGF-β signaling by SMAD7 and STAT3 signaling in HCC reported here now provides an additional rationale for co-targeting both pathways to improve the clinical spectrum of TGF-β and STAT3-based treatment options.

## Materials and methods

### Ethics statement

All animal experiments were carried out in accordance with the EU Directive 2010/63/EU for animal experiments and were approved by the government's Animal Care Committee (AZ35-9185.81/G-236/12).

### Human HCC samples

Matched pairs of HCC and adjacent non-tumour tissues from 10 patients described and analyzed for *SMAD7* expression levels before,^7^ were included in the present study. The investigation was conducted in accordance with ethical standards, with the Declaration of Helsinki and according to national and international guidelines. Informed consent was obtained from all patients, and tissue procurement was approved by the local Medical Ethics Committee (2012-293N-MA).

### Mice generation and HCC model

Albumin-SMAD7 Tg and SMAD7^fl/fl^ mice were described before.^7, 43^ Later, both mice strains were crossed with TTR-Cre mice that can control specific gene regulation in the liver,^44^ resulting in TTR-Cre-SMAD7 Tg (here: SMAD7 Tg) and TTR-Cre-SMAD7 KO and (here: SMAD7 KO). For the induction of HCC, 2-week-old mice were injected intraperitoneally with 25 mg/kg DEN (Sigma, Munich, Germany).^29^ To induce SMAD7 expression or deletion, 4-week-old mice were injected with 1 mg/mouse/day Tamoxifen (Sigma, T5648) for 5 days. Tamoxifen was dissolved in a pH neutral medium chain triglyceride (Neutralöl, Euro OTC Pharma, Bönen, Germany) at a final concentration of 10 mg/ml.^45^ Single transgenic Albumin-SMAD7 Tg and SMAD7^fl/fl^ mice were used as controls. Control mice did not show any phenotypic and functional differences to complete WT mice. At 9 months of age, mice were killed and the livers were processed for further analysis. Exclusively male mice were used for the experiments.

For analysis of tumor numbers, the livers from DEN-challenged mice were removed and washed with cold phosphate-buffered saline (PBS). Liver tumors on the surface of all lobes were counted. Diameter of the biggest tumor was measured as maximum tumor size. To analyze liver function, serum was collected and analyzed for aspartate aminotransferase and alanine aminotransferase levels. To certify significant results, group size was chosen to make sure that >8 animals remain in each group after 9 months when applying exclusion criteria or if death occurs during the experiment. No statistical method was applied to estimate sample size. All mice used for the experiments were analyzed for Smad7 recombination and expression. Exclusion criteria are described in Supplementary Figure S3. For western blotting analysis, mice from different groups were selected based on their individual *Smad7* levels in the tumor.

### Detection of recombination at DNA level

DNA from frozen tissue samples was extracted using The Invisorb Spin Tissue Mini Kit (STRATEC Biomedical AG, Birkenfeld, Germany). Detection of recombination was performed by PCR using for-primer: 5′-TGCAGACCCGGAAATTAGAC-3′ and rev-primer: 5′-TTGGATCACCATGCCAACTA-3′ (size:~290 bp) for SMAD7 KO mice as well as for-primer: 5′-TCACCTTTCCTATCAACCCC-3′ and rev-primer: 5′-CGCTCCTTGAGTTTCTTGAG-3′ for SMAD7 Tg mice (size: ~1160 bp). Two 3-month-old mice of each strain were analyzed to verify successful recombination upon Tamoxifen treatment.

### Detection of SMAD7 mRNA

RNA from frozen tissue samples was extracted by the InviTrap Spin Universal RNA Mini Kit (STRATEC Biomedical AG). Total RNA was reverse transcribed using the Omnitect Reverse Transcription Kit (Qiagen, Hilden, Germany). Quantitative RT–PCR was performed for the detection of SMAD7 mRNA (for-primer: 5′-GTGTTGCTGTGAATCTTACGG-3′ rev-primer: 5′-GATGAAGATGGGGTAACTGCT-3′). β-Actin was used as an internal control (for-primer: 5′-GTGGGCCGCCCTAGGCACCA-3′ rev-primer: 5′-TAGCCCTCGTAGATGGGCACA-3′).

### Agarose gel electrophoresis

1.2% agarose gels were prepared after heating agarose with TAE buffer (40 mM Tris-acetate, 1 mM EDTA) using a microwave. After 1 h running, gels were exposed to ultraviolet light for visualization. Densitometry was performed from RT–PCR data using the Image J software (NIH, Bethesda, MD, USA).

### Cell culture

HuH-7 cells were cultured in Dulbecco's modified Eagle's medium (Lonza, Cologne, Germany) supplemented with 10% fetal bovine serum (Invitrogen, Carlsbad, CA, USA), 4 mM L-glutamine (Lonza) and 100 U/ml penicillin/streptomycin (Biochrom KG, Berlin, Germany) in a humidified incubator at 37 °C and 5% CO_2_ atmosphere. For treatment, 10 ng/ml recombinant human cytokine IL-6 or 5 ng/ml TGF-β (both Peprotech, Hamburg, Germany) were used. Cell lines are tested regularly for mycoplasma contamination in the laboratory.

### SMAD7 adenovirus preparation and titration

SMAD7 overexpression by adenoviral (recombinant E1-deleted adenoviral vector) infection was previously described.^46^ The suitable virus multiplicity of infection=1 was determined by immunoblot after virus infection. In brief, HCC cells were seeded in six-well plates, and the different amount of virus were added and incubated with cells for 48 h. Cell lysates were collected for western blotting analysis.

### H&E staining and immunohistochemistry

Liver tissues were fixed in 4% buffered formalin solution and embedded in paraffin. Four-μm sections were stained with H&E or processed for immunohistochemistry. Antigen retrieval was performed by microwave treatment in EDTA buffer (1 mM, pH 8.0). Slides were blocked with peroxidase blocking reagent (Dako, Hamburg, Germany) for 30 min and 10% H_2_O_2_ for 15 min at room temperature. Then slides were washed with PBS and incubated with the SMAD7 antibody (1:100; ZB-8, Santa Cruz, Dallas, TX, USA), p-STAT3 antibody (1:100; 9145, Cell Signaling Technology, Danvers, MA, USA), pSmad2(S465/467) antibody (1:100, Cell Signaling Technology, 3101L), Ki67(D3B5) antibody (1:50, Cell signaling Technology, 12202S) or P21WAF1/Cip1 antibody (1:100, Sigma, P1484) at 4 °C overnight. Slides were washed with PBS twice, incubated with streptavidin-conjugated horseradish peroxidase antibody for 30 min and developed with diaminobenzidine (Sigma Aldrich, Munich, Germany) or for pSTAT3 stainings using the EnVision Detection Systems (K4065, Dako). Then slides were washes and counterstained with hematoxylin. Immunoreactivity was examined under a light microscope. Sections treated with secondary antibodies only were used as negative control.

### Immunoblot

Cells and tissues were lysed by RIPA buffer and separated by sodium dodecyl sulfate-polyacrylamide gel electrophoresis. Proteins were transferred to nitrocellulose membranes (Pierce, Rockford, IL, USA). The membranes were blocked with 5% nonfat dry milk in TBST. Primary antibodies were: SMAD7 (MAB2029, R&D Systems, Minneapolis, MN, USA), P21 (SAB4500065, Sigma Aldrich), pSMAD2 (Ser465/467), pSMAD1/3, BCL-2, BCL-XL, pSTAT3, pJNK, pc-JUN, MCL-1, cleaved caspase-3 and Actin (3101, 9520,2870, 2762, 9145, 4668, 3270,5453,9661 and 3700, Cell Signaling Technology), c-MYC, pIκBα, IL-6, TGF-β1, pERK, CyclinD1, pP38 and VEGF (sc-40, sc-1265, sc-8404, sc-130348, sc-13073, sc-753, sc-7973, and sc-507, Santa Cruz Biotechnology). Secondary antibodies were: horseradish peroxidase-linked anti-rabbit and anti-mouse antibodies (Santa Cruz Biotechnology). The membranes were developed using Supersignal Ultra (Pierce).

### IL-6 enzyme-linked immunosorbent assay

For investigation of IL-6 secretion by HuH-7 cell or in mouse serum, we used the Human IL-6 Quantikine ELISA Kit and the Mouse IL-6 Quantikine ELISA Kit (both R&D Systems), respectively. Procedures were performed according to the manufacturer's protocols. Triplicates of each sample were measured. For cell culture supernatants, two biological replicates were performed.

### Statistical analysis

Appropriate statistical methods meeting the assumptions of the tests were applied: tumor numbers and liver weights in WT, SMAD7 Tg and SMAD7 KO mice were compared by one-way analysis of variance plus Tukey's multiple comparisons test as all values in the different groups follow Gaussian distributions. Tumor sizes, relative Smad7 expression, alanine aminotransferase, aspartate aminotransferase and liver/body weight ratios in WT, SMAD7 Tg and SMAD7 KO as well as western blotting quantification were compared by non-parametric Kruskal–Wallis plus Dunn's multiple comparison tests. There was no estimate of variation within each group of data. The means of different groups differ significantly in tumor number and relative *Smad7* expression. The variance between the groups compared differed when analyzing tumor numbers but not for liver weights. Correlation of tumor numbers and *SMAD7* levels were carried out by the Spearman's rank-order coefficient method (monotonic values given, two-sided). For each analysis, *P*<0.05 indicated statistical significance. Center values are given as the mean and error bars show s.d. values. No blinding was carried out when assessing the outcome of the experiments including animal studies. No sample randomization was performed.
