# Supplementary material for: Hepatocyte-specific Smad7 deletion accelerates DEN-induced HCC via activation of STAT3 signaling in mice
Source: Oncogenesis. 2017 Jan 30;6(1):e294–. doi: 10.1038/oncsis.2016.85 (PMC5294248; doi:10.1038/oncsis.2016.85)
Supplement: Supplementary Figure Legends [file oncsis201685x1.docx]

**Supplementary figure legends**

**Supplemental figure 1.** Tamoxifen-induced CRE-dependent DNA recombination levels and liver parameters in SMAD7 Tg and SMAD7 Ko mice. (A) DNA was extracted from Wt, SMAD7 Tg and Ko mice 3 months after Tamoxifen injection. PCR products of 1160bp and 290 bp as shown in the agarose gel indicated proper DNA recombination in SMAD7 Tg and SMAD7 Ko, respectively. (B) Liver parameters were measured at the age of 9 months, before death. No significant difference in ALT and AST values was detected in the serum of mouse strains as indicated.

**Supplementary figure 2**: (A) HCC development in 9-months-old mice treated with DEN. Histological evaluation of H&E stained mouse liver tissue confirmed presence of HCC nodules. (B) Similar liver body weight ratio of 9 month old Wt, SMAD7Tg and SMAD7 Ko mice.

**Supplemental figure 3.** *Smad7* mRNA expression levels in all mice investigated in the present study, based on RT-PCR. Blue color indicates genotypes positive for SMAD7 Tg or Ko. Red labeling indicates that *Smad7* level changes are marginal as compared to Wt mice after Tamoxifen treatment. If available, both tumor and surrounding tissue were analyzed separately.

**Supplementary figure 4:** Quantification of immunoblot results shown in figure 4A, B, C. Protein levels were (A) normalized to actin and (B) normalized to Actin and *Smad7* expression of the same animals.

**Supplementary figure 5.** Immunohistochemical detection of (A) P21 and (B) Ki67 in tumor and surrounding tissue of Wt, SMAD7 Tg and SMAD7 Ko mice. Tissue sections of each strain are shown in two amplifications as indicated in the images**.** Representative pictures are shown.

**Supplementary figure 6.** Immunohistochemical detection of pSMAD2 and pSTAT3 in human HCC tissue samples (A) In human HCC tissue with low *SMAD7* expression compared to surrounding tissue positive pSMAD2 staining was detected. In 3 samples also pSTAT3 was detected. (B) In patients expressing more *SMAD7* mRNA in tumor tissue as compared to surrounding areas no pSTAT3 was detected. pSMAD2 staining was only positive in 2 patients. * indicates that these slides are not serial sections. The sections are probably cut from different angels at slightly different areas. Representative areas are shown.

**Supplementary figure 7:** IL-6 mouse serum levels and IL-6 secretion by HuH-7 cells. (A) By ELISA, IL-6 levels were detected in serum of Wt, SMAD7 Tg and SMAD7 Ko mice. Although not significant, SMAD7 Ko animals exhibit slightly elevated IL-6 levels. (B) By ELISA, IL-6 secretion was measured in HuH-7 cells naïve (ut) or infected by AdSmad7 or AdLacZ as indicated. SMAD7 overexpression did not influence IL-6 secretion. For comparison, IL-6-containing medium of IL-6-treated uninfected cells was measured.

**Supplementary figure 8:** In HuH-7 cells, TGF-β induces STAT3 phosphorylation. (A) HuH-7 cells were pretreated with TGF-β for 24h and/or stimulated with IL-6 and/or TGF-β for 1h as indicated. As expected, IL-6 induces pSTAT3 in TGF-β-pretreated and TGF-β-unstimulated cells. In TGF-β-pretreated cells, additional TGF-β stimulation for 1h results in pSTAT3 induction. (B) Quantification of immunoblot analysis was performed of 2 independent experiments.
