# Supplementary material for: Hepatocyte-specific Smad7 deletion accelerates DEN-induced HCC via activation of STAT3 signaling in mice
Source: Oncogenesis. 2017 Jan 30;6(1):e294–. doi: 10.1038/oncsis.2016.85 (PMC5294248; doi:10.1038/oncsis.2016.85)

Supplementary figure 1

A

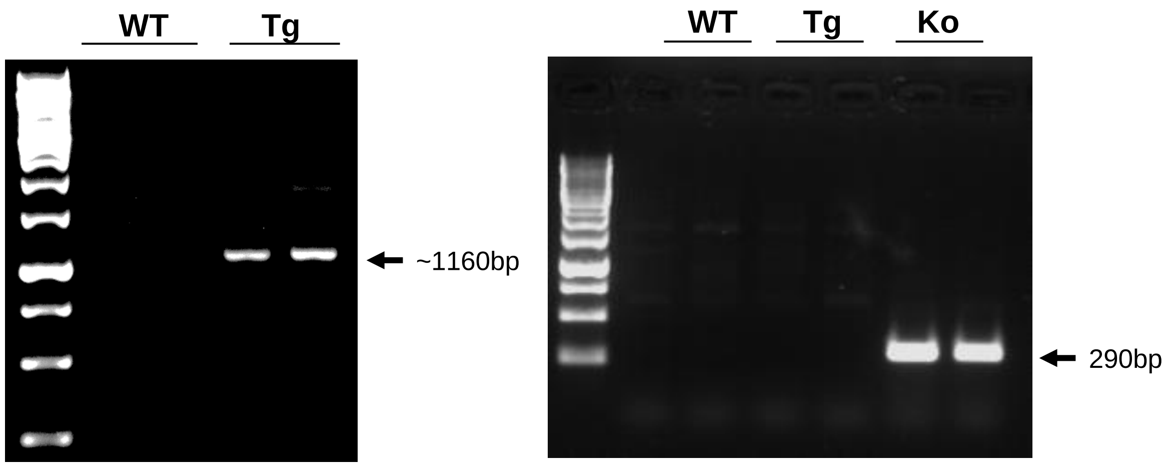

B

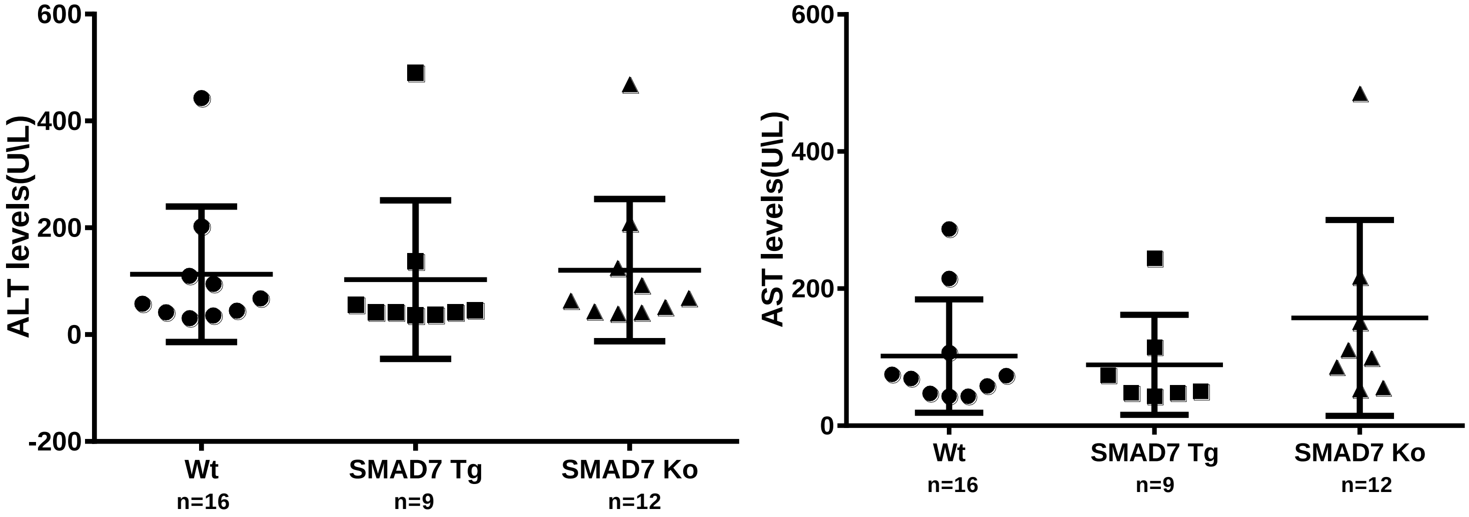

Supplementary figure 2

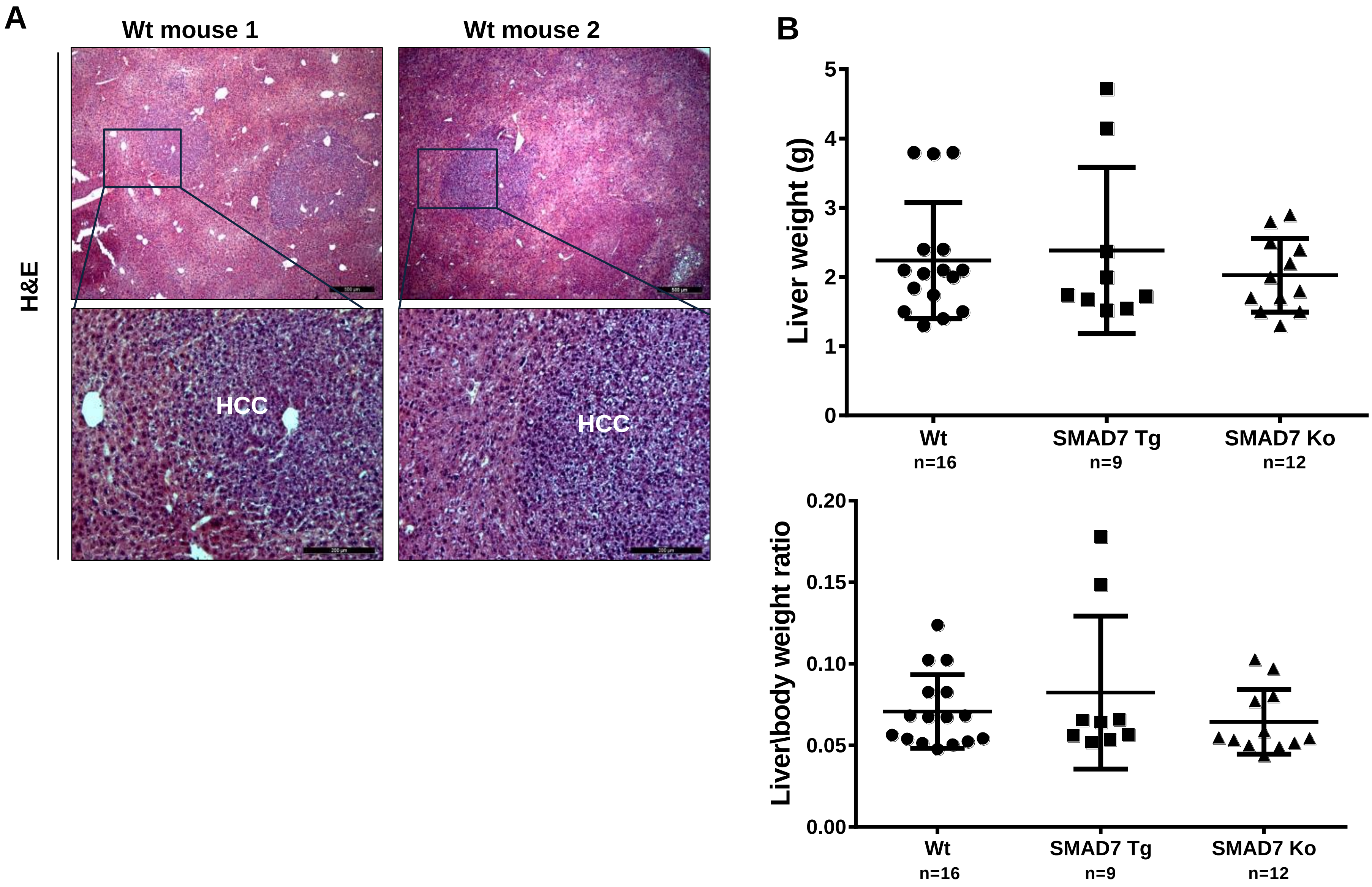

Supplementary figure 3

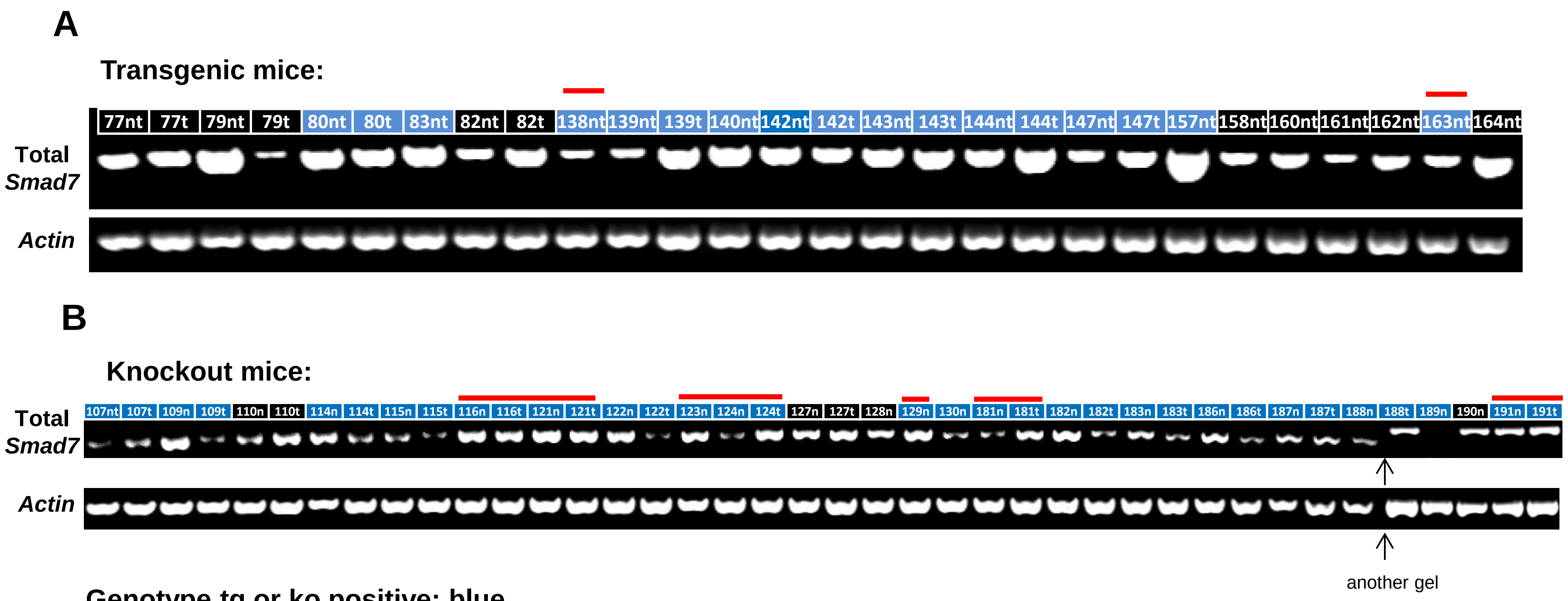

Genotype tg or ko positive: blue

Wildtype: black

Red labelled mice were excluded from the analysis of AST, ALT, tumor number, tumor size, liver weight and liver/body weight ratios based on non or adverse regulated Smad7 expression levels and/or because they did not show any tumor development for further analysis
All mice were included in the analysis shown in figure 3.

Supplementary figure 4A **Normalized to actin and Smad7**

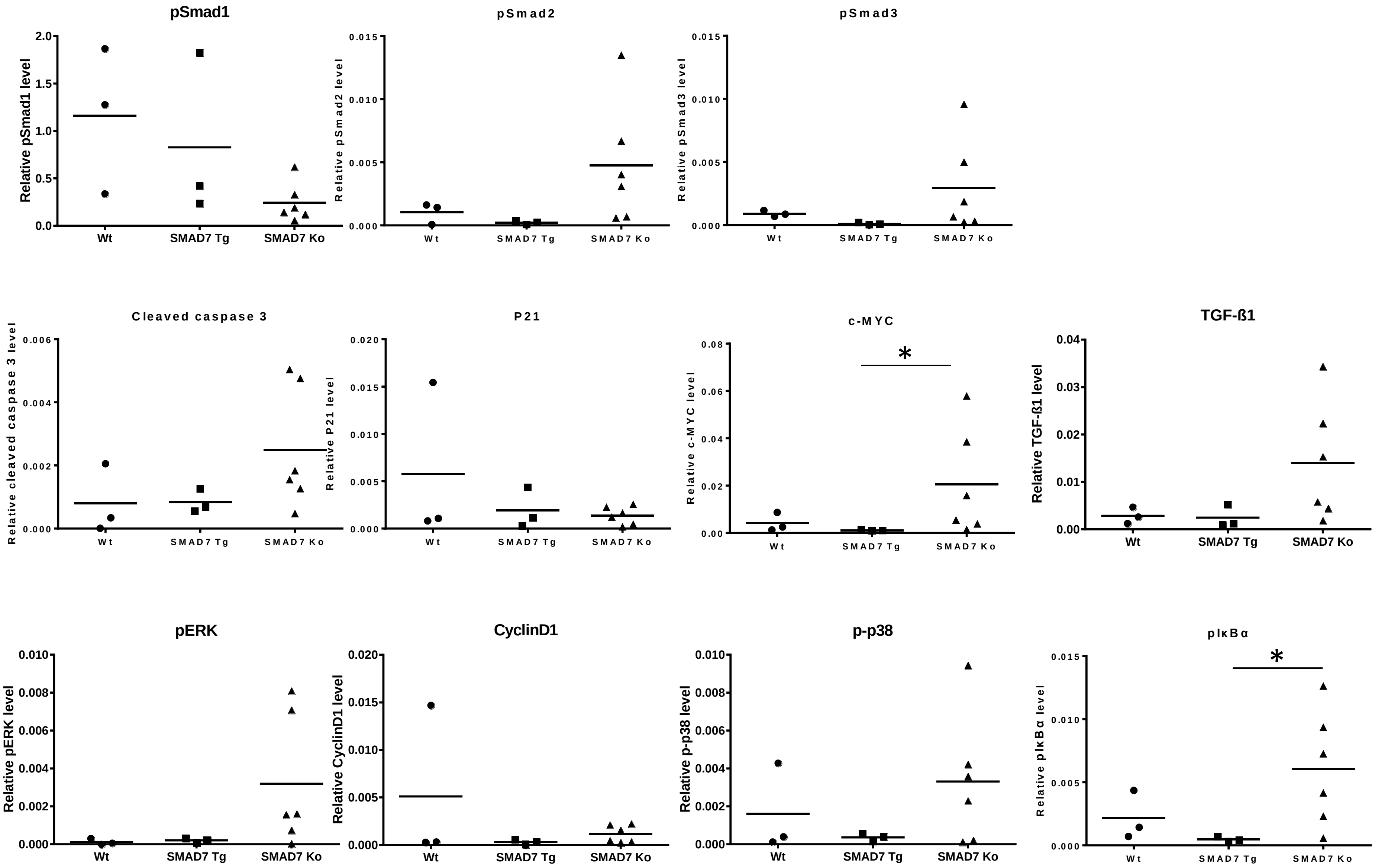

Supplementary figure 4 A **Normalized to actin and Smad7**

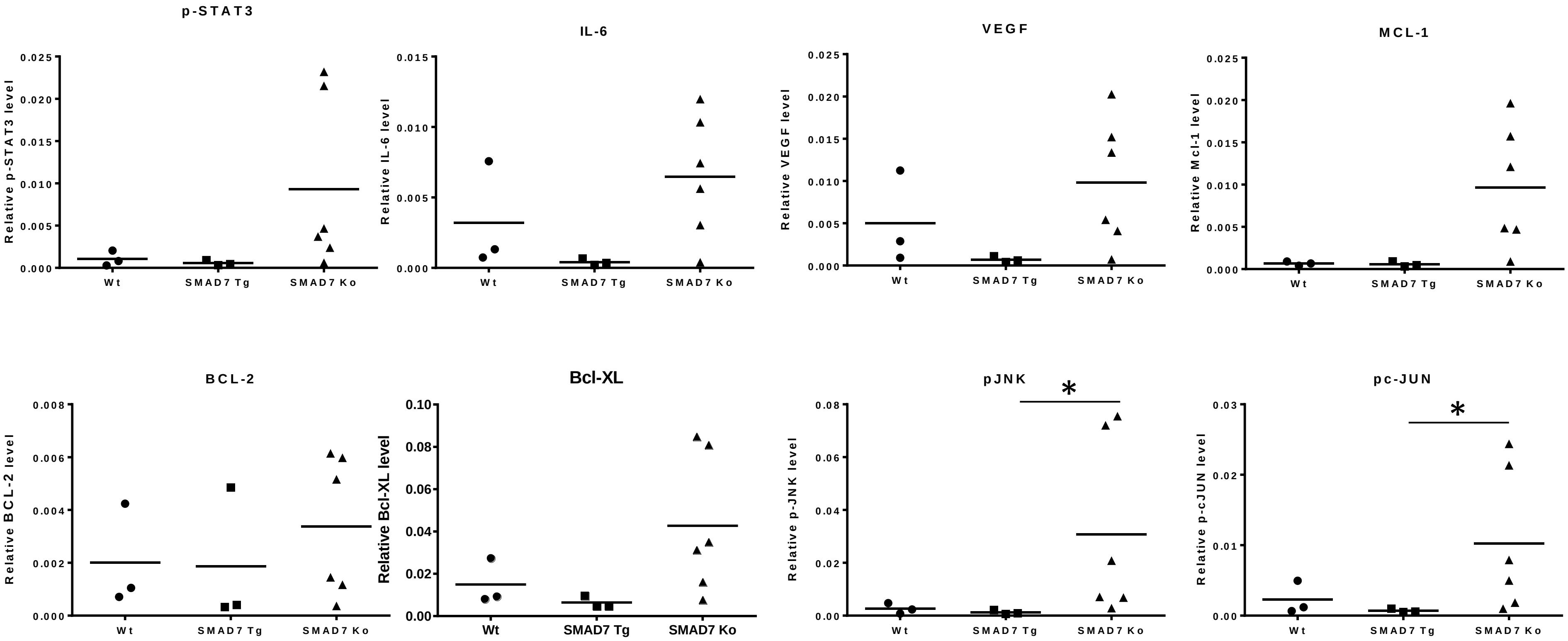

Supplementary figure 4 B **Normalized to actin**

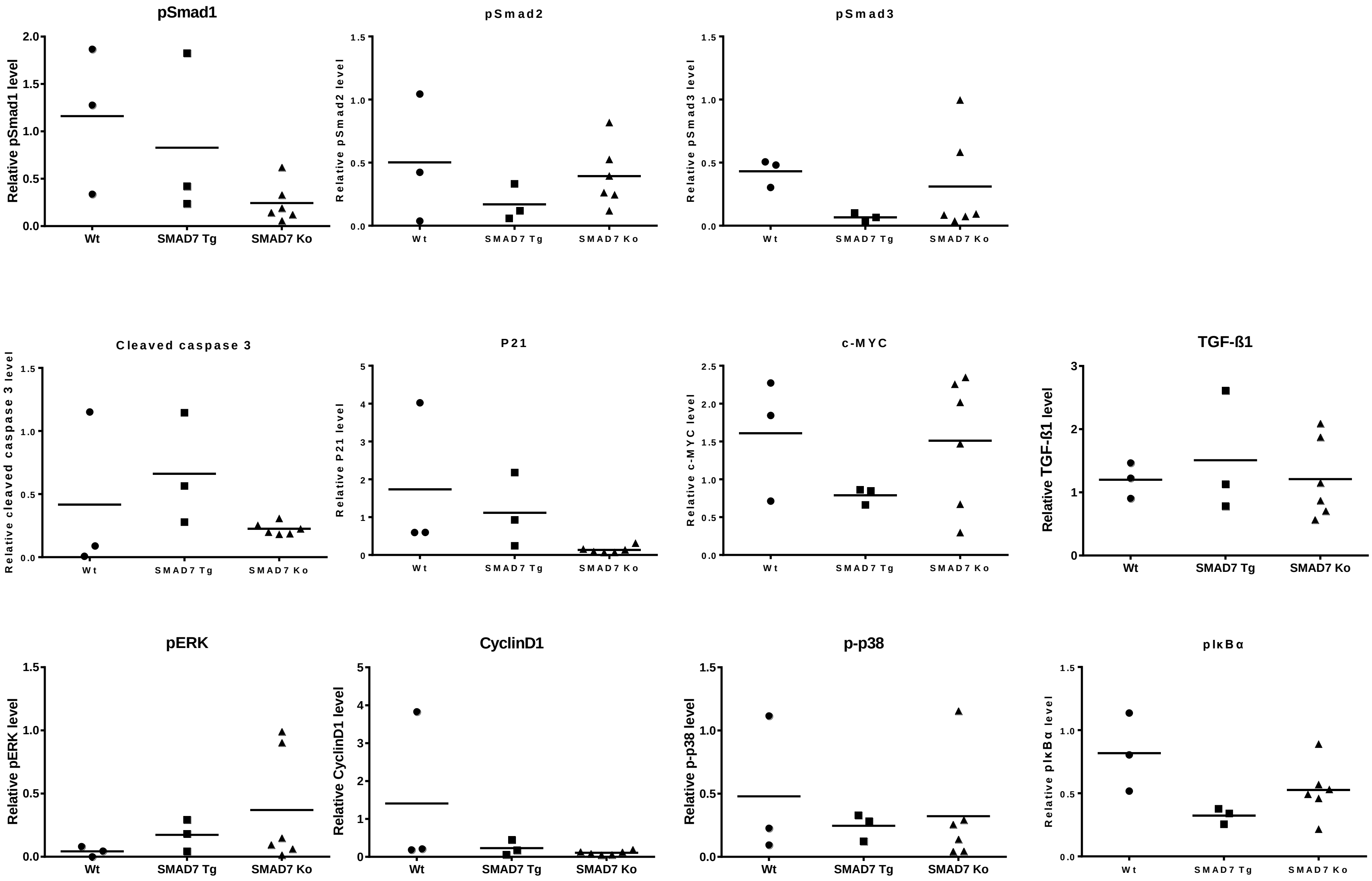

Supplementary figure 4 B **Normalized to actin**

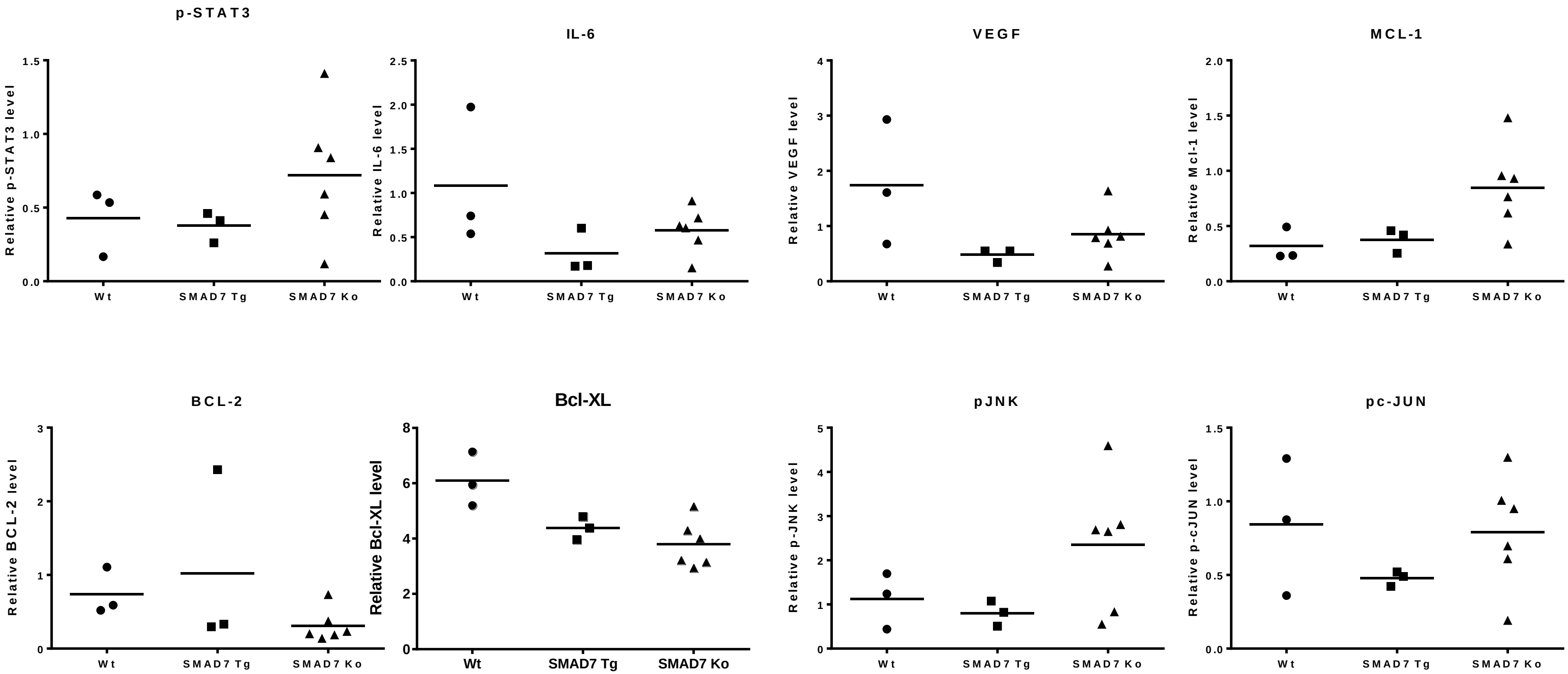

Supplementary figure 5

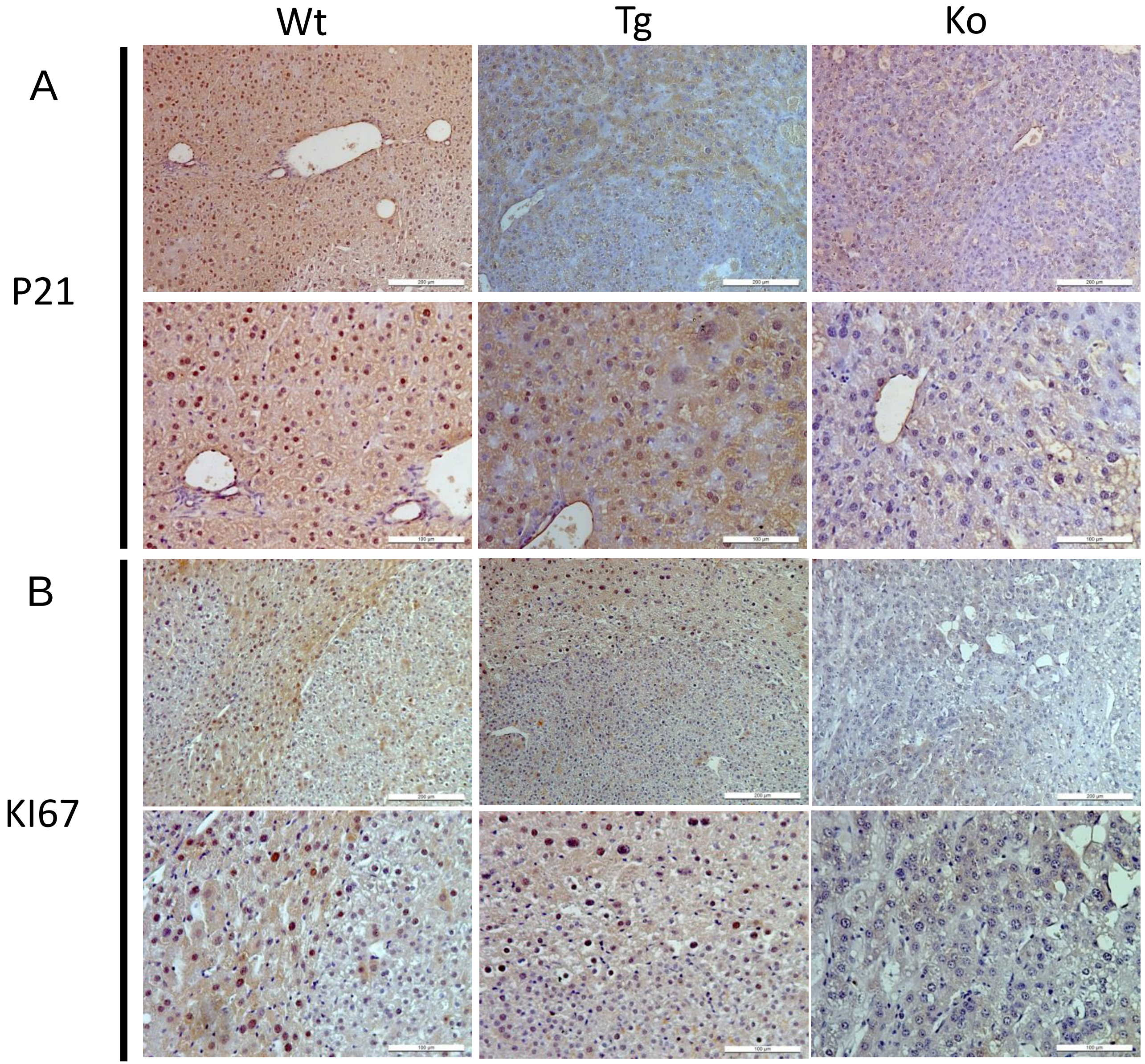

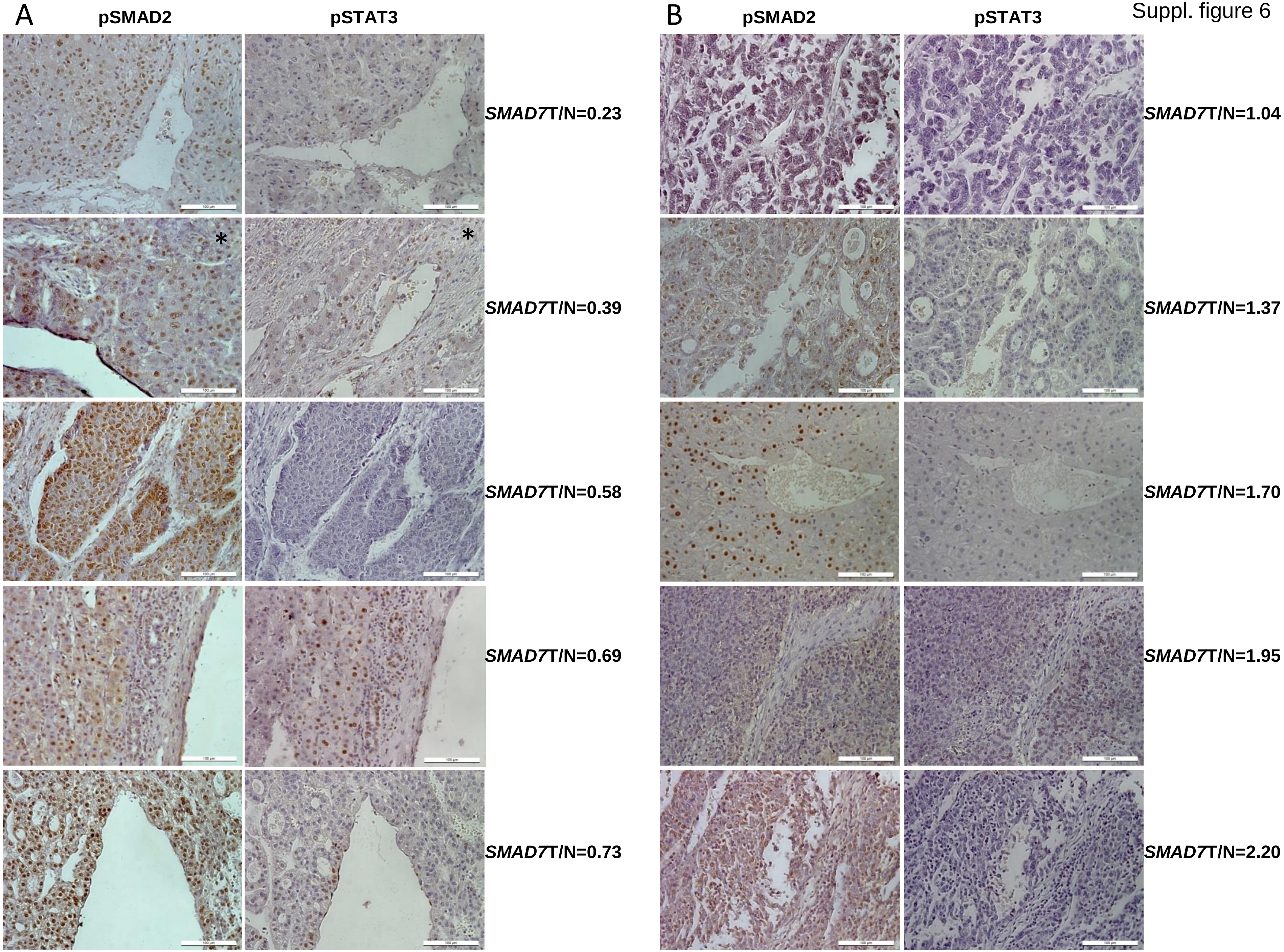
A
pSMAD2
pSTAT3
SMAD7T/N=0.23
SMAD7T/N=0.39
SMAD7T/N=0.58
SMAD7T/N=0.69
SMAD7T/N=0.73
*
*
B
pSMAD2
pSTAT3
SMAD7T/N=1.04
SMAD7T/N=1.37
SMAD7T/N=1.70
SMAD7T/N=1.95
SMAD7T/N=2.20

Supplementary figure 7

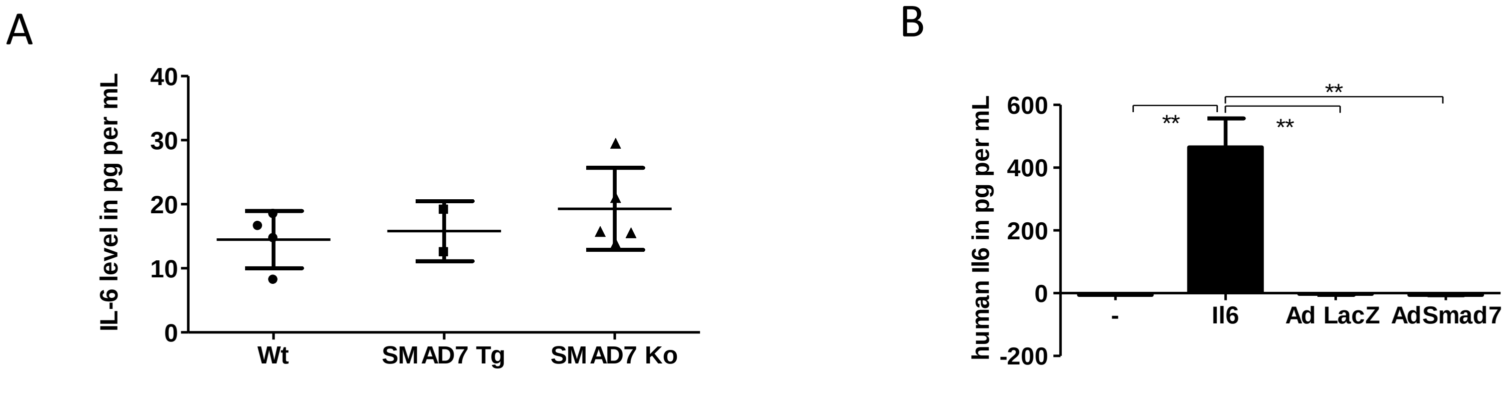

Supplementary figure 8

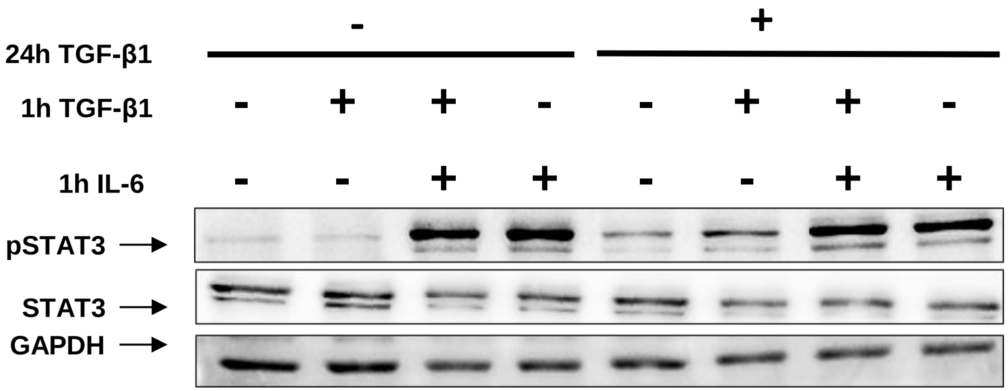

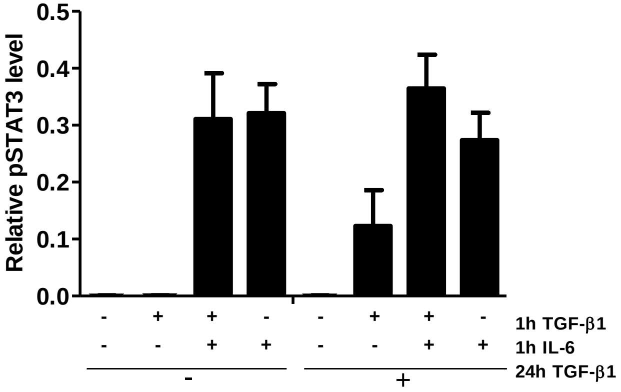

Supplement: Supplementary Figures [file oncsis201685x2.pdf]
